# BMP-9 regulates the osteoblastic differentiation and calcification of vascular smooth muscle cells through an ALK1 mediated pathway

**DOI:** 10.1111/jcmm.12373

**Published:** 2014-10-09

**Authors:** Dongxing Zhu, Neil Charles Wallace Mackenzie, Catherine M Shanahan, Rukshana C Shroff, Colin Farquharson, Vicky Elizabeth MacRae

**Affiliations:** a The Roslin Institute and Royal (Dick) School of Veterinary Studies, The University of EdinburghMidlothian, UK; b BHF Centre, Cardiovascular Division, King's College LondonLondon, UK; c Nephrology Unit, Great Ormond Street HospitalLondon, UK

**Keywords:** vascular calcification, vascular smooth muscle cells, BMP-9, ALK1

## Abstract

The process of vascular calcification shares many similarities with that of physiological skeletal mineralization, and involves the deposition of hydroxyapatite crystals in arteries. However, the cellular mechanisms responsible have yet to be fully explained. Bone morphogenetic protein (BMP-9) has been shown to exert direct effects on both bone development and vascular function. In the present study, we have investigated the role of BMP-9 in vascular smooth muscle cell (VSMC) calcification. Vessel calcification in chronic kidney disease (CKD) begins pre-dialysis, with factors specific to the dialysis milieu triggering accelerated calcification. Intriguingly, BMP-9 was markedly elevated in serum from CKD children on dialysis. Furthermore, *in vitro* studies revealed that BMP-9 treatment causes a significant increase in VSMC calcium content, alkaline phosphatase (ALP) activity and mRNA expression of osteogenic markers. BMP-9-induced calcium deposition was significantly reduced following treatment with the ALP inhibitor 2,5-Dimethoxy-*N*-(quinolin-3-yl) benzenesulfonamide confirming the mediatory role of ALP in this process. The inhibition of ALK1 signalling using a soluble chimeric protein significantly reduced calcium deposition and ALP activity, confirming that BMP-9 is a physiological ALK1 ligand. Signal transduction studies revealed that BMP-9 induced Smad2, Smad3 and Smad1/5/8 phosphorylation. As these Smad proteins directly bind to Smad4 to activate target genes, siRNA studies were subsequently undertaken to examine the functional role of Smad4 in VSMC calcification. Smad4-siRNA transfection induced a significant reduction in ALP activity and calcium deposition. These novel data demonstrate that BMP-9 induces VSMC osteogenic differentiation and calcification *via* ALK1, Smad and ALP dependent mechanisms. This may identify new potential therapeutic strategies for clinical intervention.

## Introduction

Arterial medial calcification (AMC), a hallmark of disease in patients with end-stage kidney disease, is highly correlated with elevated serum phosphate levels and cardiovascular mortality [1,2]. AMC is recognized as an active, tightly regulated process, sharing many similarities with physiological bone formation [3], and involves the deposition of hydroxyapatite crystals in arteries. Indeed vascular smooth muscle cells (VSMCs), the predominant cell type involved in AMC, can undergo transdifferentiation to a chondrocytic, osteoblastic and osteocytic phenotype in a calcified environment [3,4].

Bone morphogenetic proteins (BMPs) constitute a group of signalling factors that orchestrate embryonic patterning in development and contribute to post-natal tissue remodelling. Over 20 identified BMP ligands are recognized by type I and type II serine-threonine kinase BMP receptors [5]. Ligand binding induces constitutively active BMP type II receptors to transphosphorylate BMP type I receptors, which in turn phosphorylate the intracellular BMP effector proteins, Smad 1/5/8. Signalling through Smad1/5/8 activation mediates the principal effects of BMPs, although activation of additional pathways may further refine cellular effects [6].

Bone morphogenetic proteins are vital regulators in orthotopic bone formation, and their localization at sites of vascular calcification raises the question of their role in this pathological process. The concept that BMPs mediate vascular calcification is supported by the knowledge that smooth muscle-targeted overexpression of BMP-2 accelerates vascular calcification in atherogenic mice [7]. Furthermore, loss of matrix Gla protein (MGP), an endogenous BMP inhibitor, causes extensive calcification of elastic and muscular arteries [8], suggesting that regulation of BMP activity is essential for maintaining a normal vessel media.

As one of the least studied BMPs, BMP-9 has to date been shown to regulate the cholinergic phenotype of embryonic basal forebrain cholinergic neurons [9], controlling iron homoeostasis [10] and modulating key enzymes of lipid metabolism [11]. A number of studies have also established BMP-9 as a key regulator of angiogenesis in endothelial cells [12–14]. Of direct relevance to this study, BMP-9 has recently been identified as one of the most osteogenic BMPs, regulating several downstream targets during BMP-9-induced osteoblast differentiation including Runx2, osteocalcin and Tissue-nonspecific alkaline phosphatase (ALP) [15]. However, the role of BMP-9 in regulating the phenotypic transdifferentiation of VSMCs during calcification is unknown and warrants investigation. Therefore, we have undertaken clinical analyses in conjunction with *in vitro* VSMC calcification studies to identify BMP-9 and the BMP signalling pathway as a potential therapeutic target for modifying vascular calcific disease.

## Materials and methods

### Ethics statement

For clinical studies, informed written consent was obtained from all parents or caregivers and children, where appropriate. The study was approved by the local research ethics committee. All animal experiments were approved by The Roslin Institute's Animal Users Committee and the animals were maintained in accordance with Home Office guidelines for the care and use of laboratory animals.

### Clinical samples

Blood samples were obtained from 10 children in pre-dialysis chronic kidney disease (CKD) stage V (estimated glomerular filtration rate <15 ml/min./1.73 m^2^) and 10 children on haemodialysis. Samples were collected at a routine clinic visit or before a mid-week session of haemodialysis. To keep the groups free of confounding pro-atherosclerotic risk factors, children with underlying inflammatory disorders, vasculitis, diabetes, dyslipidaemia or smokers were excluded. Serum biochemical parameters (calcium, phosphate and high sensitivity C-Reactive Protein (hs-CRP)) and BMP-9 concentrations (R&D Systems, Abingdon, UK) were determined.

### Materials

Recombinant mouse BMP-9 and Activin receptor-like kinase-1 fusion protein (ALK1-Fc) were from R&D Systems. The ALP inhibitor 2,5-Dimethoxy-*N*-(quinolin-3-yl) benzenesulfonamide (DBS) was from Merk KGaA (Darmstadt, Germany). Antibodies to phosphorylated Smad1/5/8, Smad2, Smad3, Akt (ser 473) and Erk1/2 Map kinase (Thr202-/Tyr204) and to total Smad1/5/8, Smad2, Smad3, Smad4, Akt and Erk1/2 Map kinase were from Cell Signaling Technology (Beverly, MA, USA). Antibodies to anti-alpha-smooth muscle actin (SMA) and anti-CD31 were from Sigma-Aldrich (Poole, UK) and Abcam (Cambridge, UK), respectively. α-MEM medium, Fetal Bovine Serum (FBS), Gentamicin, Alexa Fluor^@^488 goat-antimouse and Alexa Fluor^@^594 goat-anti rabbit antibodies were obtained from Invitrogen (Paisley, UK). Collagenase type II was from Worthington Biochemical Corporation (Lakewood, NJ, USA). Laminin was from Sigma-Aldrich. Tissue culture flasks were from Greiner Bio-one (Frickenhausen, Baden-Wurttemberg, Germany).

### Preparation of primary murine VSMCs

Primary VSMCs were prepared from 5-week old wild-type (WT) C57BL/6 mice and cultured in growth medium as previously described [4,16]. Briefly, after careful removal of adventitia, the aorta was cut open to expose endothelial layer under a dissection microscope. Tissues from eight animals were pooled together and incubated with 1 mg/ml trypsin for 10 min to remove any remaining adventitia and endothelium. After a further overnight incubation at 37°C in a humidified atmosphere of 95% air/5% CO_2_ in growth medium consisting of α-MEM supplemented with 10% FBS and 1% gentamicin, tissues were digested with 425 U/ml collagenase type II for 5 hrs. Medial cells were released and cell suspension was centrifuged at 2000 × g for 5 min. The cell pellet was washed and re-suspended in the above mentioned growth medium. Isolated VSMCs were cultured with growth medium for two passages in T25 tissue culture flasks coated with 0.25 μg/cm^2^ laminin to promote maintenance of the contractile differentiation state [17].

### Induction of calcification

Primary VSMCs were seeded in growth medium at a density of 1.5 × 10^4^/cm^2^ in multi-well plates. Calcification was induced as previously described [16]. In brief, cells were grown to confluence (day 0) and switched to calcification medium, which was prepared by adding inorganic phosphate (a mixture of NaH_2_PO_4_ and Na_2_HPO_4_, pH 7.4) (Sigma-Aldrich), to reach a final concentration of 3 mM phosphate. VSMCs were incubated for up to 14 days in 95% air/5% CO_2_ and medium was changed every third/fourth day. Recombinant mouse BMP-9, ALK1-Fc or 3 μM ALP inhibitor was added at day 0.

### Determination of calcification

Calcium deposition was quantified as previously described [16]. Briefly, cells were rinsed twice with PBS and decalcified with 0.6 N HCL at room temperature for 24 hrs. Free calcium was determined colorometrically by a stable interaction with phenolsulphonethalein using a commercially available kit (Randox Laboratories Ltd., County Antrim, UK) and corrected for total protein concentration (Bio-Rad Laboratories Ltd, Hemel Hempstead, UK).

Calcium deposition was also evaluated by alizarin red staining as previously described [16,18]. Cells were washed twice with PBS, fixed in 4% paraformaldehyde for 5 min at 4°C, stained with 2% alizarin red (pH 4.2) for 5 min at room temperature and rinsed with distilled water.

### Alkaline phosphatase activity

Alkaline phosphatase activity from whole cell lysates was assayed as previously described [4,16]. Cells were washed with PBS twice, solubilized with 0.2% Triton X-100 in 0.9% NaCl and assayed for ALP activity. Enzyme activity was determined by measuring the cleavage of 10 mM p-nitrophenyl phosphate (pNPP) at 410 nm using a commercially available kit (Thermo Trace, Melbourne, Australia). Values were normalized to total protein levels, as assessed by the Bio-Rad protein assay reagent (Bio-Rad Laboratories, Hertfordshire, UK), and gamma globulin was used as standard. Total ALP activity was expressed as nmoles pNPP hydrolysed/min./mg protein.

### Analysis of gene expression

RNA was extracted using RNeasy total RNA (Qiagen Ltd, Crawley, West Sussex, UK), according to the manufacturer's instructions. RNA was reverse transcribed and specific cDNAs were semi-quantified by end-point PCR or quantified by real-time PCR using the SYBR green detection method as previously reported [4,16]. Primers were obtained from Eurofins MWG Biotech (Ebersberg, Germany), Qiagen and Primer Design (Southampton, UK), with available sequences provided in the online Data Supplement (Table S1).

### Western blotting

Cultured cells were harvested in RIPA buffer (Sigma-Aldrich) containing ‘complete’ protease inhibitor cocktail according to manufacturer's instructions (Roche, East Sussex, UK). Western blotting was performed as previously described [19]. Nitrocellulose membranes were probed overnight at 4°C with anti-sclerostin antibody (R&D Systems) or BMP-9 primary antibody [Abcam, 1:1000 dilution in 5% bovine serum albumin (BSA)], washed in TBST and incubated with anti-goat or anti rabbit IgG-peroxidase respectively (DAKO, Glostrup, Denmark) for 1 hr (1:1000 dilution in 5% milk). The immune complexes were visualized using enhanced chemi-luminescence (ECL) (GE Healthcare, Buckinghamshire, UK). Membranes were then washed in ‘stripping buffer’ (Pierce, Rockford, Il, USA) and re-probed for 1 hr for β-actin expression (1:5000 dilution in 5% milk; anti β-actin clone AC15; Sigma-Aldrich).

### Cell signalling immunoblotting

Vascular smooth muscle cells were grown to confluence and serum starved for 24 hrs. Cells were treated with 0–50 ng/ml recombinant mouse BMP-9 for 30 min or 50 ng/ml BMP-9 for 0–60 min (R&D Systems). Cells were lysed in PhosphoSafe extraction buffer (Merck Biosciences Ltd, Nottingham, UK) containing ‘Complete’ protease inhibitor cocktail (Roche) according to manufacturer's instructions for cell signalling studies. Western blotting was performed as previously described [4]. Nitrocellulose membranes were probed overnight at 4°C with the relevant primary rabbit antibody (phospho-Smad1/5/8, phospho-Smad2, phospho-Smad3, total Smad2, total Smad3, Smad4, phospho-Akt (ser 473), total Akt, phospho-Erk1/2 Map kinase (Thr202-/Tyr204) and total Erk1/2 Map kinase or total Smad1/5/8). The membranes were then incubated with anti rabbit IgG-peroxidase (Cell Signalling Technology) for 1 hr (1:1000 dilution in 5% milk). The immune complexes were visualized using enhanced chemi-luminescence (ECL) Western Blotting Detection System (GE Healthcare, Buckinghamshire, UK).

### Transfection assays

Vascular smooth muscle cells were seeded in 12-well plates at a density of 80,000 cells/well and transfected with Allstars Negative Control siRNA (20 nM; Scrambled siRNA (Qiagen Ltd)) or *Smad4* siRNA (20 nM; Qiagen Ltd) with Hyperfect transfection reagent (Qiagen Ltd), according to manufacturer's instructions. Cells were used for experiments 48 or 96 hrs after transfection. The knock-down efficiency of Smad4 was verified by qRT-PCR and western blotting. For long-term VSMC calcification assay, cells were re-transfected at day 4 and cultured in the presence of the calcification medium for up to 9 days.

### Fluorescent immunocytochemical staining

Vascular smooth muscle cells were seeded on glass coverslips in 12-well plates at a density of 50,000 cells/well. Following confluence, VSMCs were serum-restricted for 24 hrs and stimulated with 0–50 ng/ml recombinant mouse BMP-9 for 30 min. Cells were fixed with 4% paraformaldehyde and washed with PBS. The fixed cells were permeabilized with 0.3% Triton X-100 (Sigma-Aldrich) and incubated with anti-alpha-SMA, anti-CD31, or anti-phospho-Smad1/5/8 antibody overnight at 4°C. After washing, cells were incubated with Alexa Fluor@488 goat-anti mouse antibody or Alexa Fluor@594 goat-anti rabbit antibody for 1 hr in the dark. Glass coverslips were mounted onto slides with Prolong®Gold Anti-Fade Reagent contained DAPI (Invitrogen). Fluorescence signal was detected under a Leica fluorescence microscope (Milton Keynes, UK).

### Flow cytometric analysis

Vascular smooth muscle cell suspensions were obtained by trypsinisation (0.25% Trypsin-EDTA, Invitrogen), and washed with PBS twice. Cells were incubated with isotype control or anti-CD31 antibody (1:100 dilution, Abcam) for 30 min at room temperature. Cells were washed and incubated with Alexa Fluor@594 goat-anti rabbit antibodies (1:1000 dilution, Invitrogen) for 30 min in the dark. After wash, cells were analysed with a BD Fortessa (BD Biosciences. Oxford, UK). Data were evaluated using FlowJo v7.5.5 (Tree Star, Ashland, OR, USA).

### BMP-9 ELISA

Human serum of 100 μl was used to measure BMP-9 concentrations using the human BMP-9 DuoSet Kit (R&D Systems) according to manufacturer's instructions. BMP-9 recombinant protein standards ranging from 0 to 1000 pg/ml were prepared in 1% BSA in PBS. The plate was read at a dual wave length of 450 and 540 nm using a BioTek plate reader (BioTek, Bedfordshire, UK). BMP-9 concentration was determined by reading from the standard curve, which was created by Gen5 software (BioTek) using a four parameter logistic (4-PL) curve-fit.

### Statistics

Data are presented as the means ± SEM. Statistical analysis was determined by General Linear Model Analysis incorporating pairwise comparisons, the Student's *t*-test using Minitab 16 (Minitab Inc, Coventry, UK). *P* < 0.05 was considered to be significant. *N* = x indicates the number of different wells of a representative cell culture experiment. Each cell culture experiment was repeated at least twice.

## Results

### Increased serum BMP-9 in CKD dialysis patients

**Fig. 1:**
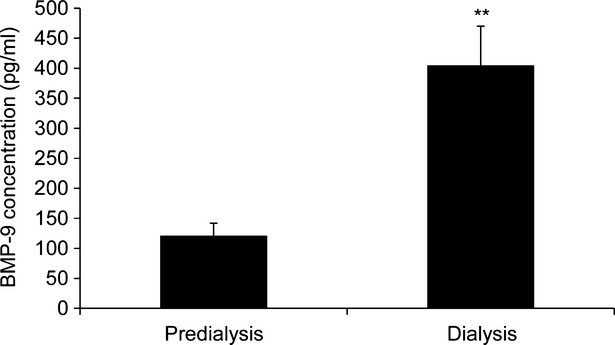
Assessment of bone morphogenetic protein (BMP-9) in serum from CKD dialysis patients. BMP-9 concentrations (pg/ml) in pre-dialysis (*n* = 10) and dialysis (*n* = 9) serum from children with CKD.

BMP-9 was markedly elevated in serum from children on haemodialysis (234% increase compared to pre-dialysis CKD; *P* < 0.001; Fig. 1). No significant differences in calcium, phosphate or calcium × phosphate product were noted (Table 1). Furthermore, no correlation between BMP-9 concentration and high sensitivity C-Reactive Protein (hs-CRP) were seen (Table 1). These data are the first to show that BMP-9 is elevated in dialysis patients.

**Table 1:** Clinical and biochemical features of the pre-dialysis and dialysis groups

Serum	Ca (mmol/l)	PO_4_ (mmol/l)	Ca × PO_4_	hs-CRP (mg/l)
Pre-dialysis (*n* = 10)	2.40 (0.04)	1.55 (0.06)	3.71 (0.17)	
Dialysis (*n* = 9)	2.46 (0.02)	1.64 (0.05)	4.04 (0.13)	13.48 (3.25)

### Up-regulation of BMP-9 during the VSMC calcification process

**Fig. 2:**
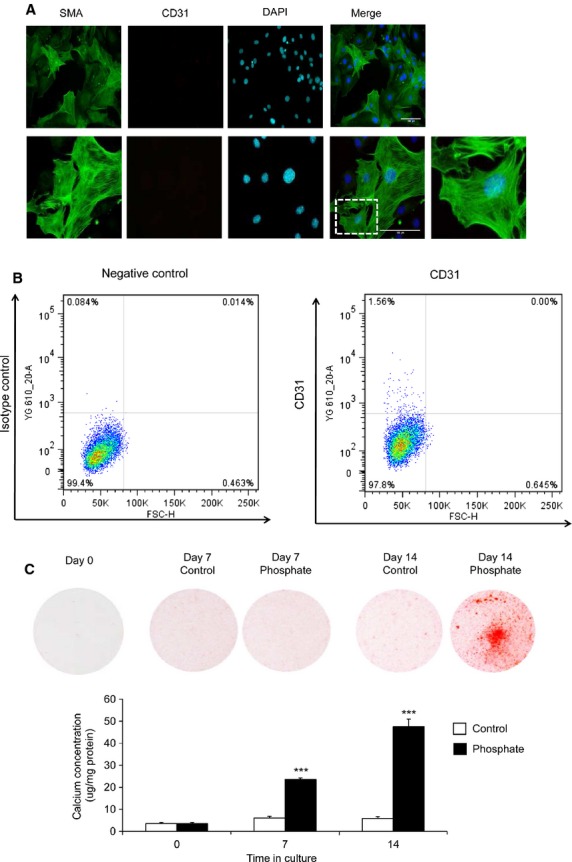
High P_i_ induces the calcification of VSMCs. (**A**) Immunofluorescence staining of murine primary VSMCs demonstrates positive staining for the smooth muscle cell marker smooth muscle actin (SMA; Green) and negative staining for the endothelial cell marker, CD31. (**B**) FACs analysis further confirmed only 1.56% of isolated cells positively stained for CD31. (**C**) Alizarin red staining and Quantification of calcification by HCL leaching (*n* = 3) in VSMCs cultured with high phosphate (3 mM P_i_; filled bar) or control (1 mM P_i_; white bar) medium (*n* = 5). Results are presented as mean ± SEM. ***P < 0.001 compared with control; scale bar = 100 μm.

We confirmed that murine primary VSMCs isolated in the present study were free from endothelial contamination. Cells were negative for the endothelial cell marker, CD31 (Fig. 2A). In addition, cells showed positive staining for the smooth muscle cell marker SMA (Green; Fig. 2A). FACs analysis further confirmed only 1.56% of isolated cells positively stained for CD31 (Fig. 2B). Since AMC is highly correlated with elevated serum phosphate levels, VSMCs were cultured in growth medium containing high P_i_ (3 mM P_i_). High P_i_ induced a significant increase in VSMC calcium deposition (determined by HCL leaching and alizarin red staining) at day 7 and day 14, compared to cells cultured in control medium (1 mM P_i_; *P* < 0.001; Fig. 2C).

**Fig. 3:**
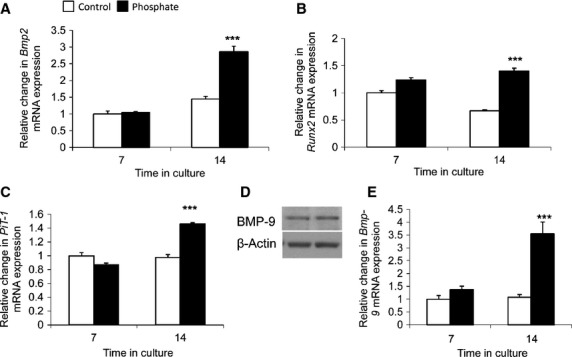
Up-regulated bone morphogenetic protein (*BMP-9*) expression during the calcification process of VSMCs. VSMCs were cultured with high phosphate (3 mM P_i_; filled bar) or control (1 mM P_i_; white bar) medium (*n* = 5) by 7 and 14 days. Fold change in the mRNA expression of (**A**) *Bmp2*, (**B**) *Runx2* and (**C**) *PiT-1*. (**D**) BMP-9 protein was expressed by VSMCs at 0 day. (**E**) Fold change in the mRNA expression of *BMP-9*. Results are presented as mean ± SEM. ***P < 0.001 compared with control.

Consistent with previous studies [4,16,20,21,19], high P_i_ induced osteogenic transition of VSMCs, as demonstrated by significantly up-regulated expression of *Bmp2* (2.0-fold, *P* < 0.001; Fig. 3A), *Runx2* (2.1-fold, *P* < 0.001; Fig. 3B) and *PiT-1* (1.7-fold, *P* < 0.001; Fig. 3C) at 14 days. Interestingly, while a significant increase in VSMC calcification was observed after 7 days of treatment with high phosphate (Fig. 2C), increased expression of osteogenic markers was only observed after 14 days of treatment. High phosphate may therefore initially regulate VSMC calcification through additional mechanisms to the osteogenic differentiation of vascular cells, such as loss of calcification inhibition, matrix degradation and apoptosis [2].

Having established basal levels of BMP9 expression in VSMCS (Fig. 3D), BMP-9 mRNA expression (3.3-fold, *P* < 0.001; Fig. 3E) was shown to be significantly increased at 14 days in VSMCs cultured in calcifying medium. These data suggest that BMP-9 may be actively involved in the calcification process.

### BMP-9 induces osteogenic differentiation of VSMCs and promotes high P_i_-induced calcification

**Fig. 4:**
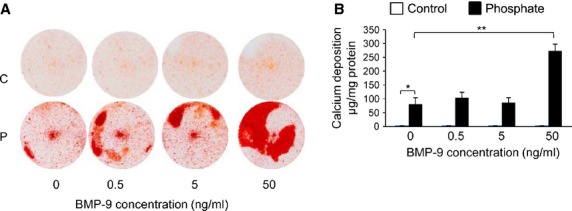
Bone morphogenetic protein (BMP-9) promotes the calcification of VSMCs. VSMCs were incubated with BMP-9 (0.5–50 ng/ml) in high phosphate (P) (3 mM P_i_) or control (C) (1 mM P_i_) medium for 9 days. Calcium content was (**A**) visualized with alizarin red staining and (**B**) quantified by HCL leaching (μg/mg protein; *n* = 5). Results are presented as mean ± SEM. *P < 0.05; **P < 0.01; compared with 0 ng/ml BMP-9 treatment or control.

**Fig. 5:**
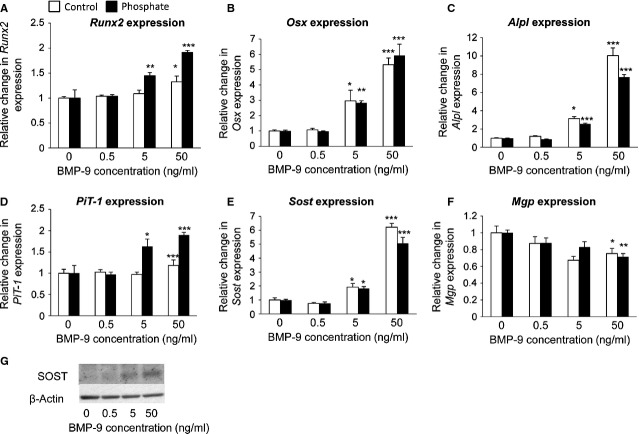
Effect of bone morphogenetic protein (BMP-9) treatment on the osteogenic marker expression. Fold change in the mRNA expression of osteogenic/osteocytic markers (**A**) *Runx2*, (**B**) *Osx*, (**C**) *ALP*, (**D**) *PiT-1*, (**E**) *Mgp* and (**F**) *Sost* (*n* = 4). Results are presented as mean ± SEM. *P < 0.05; **P < 0.01; ***P < 0.001 compared with corresponding 0 ng/ml BMP-9 treatment. (**G**) Sclerostin protein expression was increased following BMP-9 treatment.

To test whether BMP-9 directly regulates the osteogenic differentiation and calcification of VSMCs, we treated cells with 0.5–50 ng/ml BMP-9 in the presence or absence of high P_i_ medium for up to 9 days. In the presence of high P_i_, a significant increase in calcium deposition was observed following BMP-9 treatment at 50 ng/ml, as determined by alizarin red staining and HCL leaching (3.4-fold; *P* < 0.01; Fig. 4A and B). However, no increase in calcification was observed under control P_i_ conditions (Fig. 4A and B). In the presence of both high P_i_ and control conditions, a minimum concentration of 50 ng/ml BMP-9 treatment induced a significant increase in the mRNA expression of the osteogenic markers *Runx2*, *Osterix*, *Akp2*, *PiT-1* and *Sost* (*P* < 0.05; Fig. 5A–E). Furthermore, a concomitant reduction in the mRNA expression of the mineralization inhibitor *Mgp* was observed following treatment of VSMCs with 50 ng/ml BMP-9 (*P* < 0.05; Fig. 5F). Comparable changes in sclerostin protein expression were also observed following treatment of VSMCs with 50 ng/ml BMP-9 (Fig. 5G). These data suggest that BMP-9 directly induces osteogenic differentiation of VSMCs, and subsequently increases the susceptibility of VSMCs to calcification in the presence of high P_i_.

**Fig. 6:**
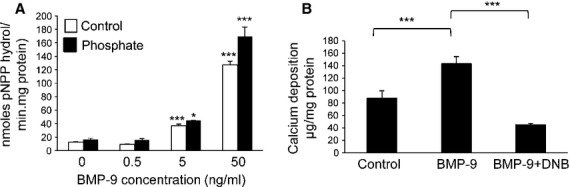
Critical role of alkaline phosphatase (ALP) in bone morphogenetic protein (BMP-9) induced VSMC calcification. (**A**) Quantification of alkaline phosphatase activity (mean moles pNPP hydrol/min/mg protein in VSMCs incubated with BMP-9 (0.5–50 ng/ml) in high phosphate (3 mM P_i_; filled bar) or control (1 mM P_i_; white bar) medium for 4 days (*n* = 5). (**B**) Calcium content was quantified by HCL leaching (μg/mg protein) in VSMCs incubated with BMP-9 (50 ng/ml) in high phosphate (3 mM P_i_) in the presence/absence of the ALP inhibitor DNB (3 μM; *n* = 5). Results are presented as mean ± SEM. *P < 0.05; ***P < 0.001 compared with 0 ng/ml BMP-9 treatment or control.

In addition, treatment with BMP-9 for 4 days dose-dependently induced ALP activity (2.7-fold at 5 ng/ml; *P* < 0.001; Fig. 6A) in both high P_i_ and low P_i_ conditions. Interestingly, co-treatment of BMP-9 (50 ng/ml) with the ALP inhibitor DNB (3 μM) significantly reduced the pro-calcificatory effects of BMP-9 (68%; *P* < 0.001; Fig. 6B).

### BMP-9 signals through the ALK1 receptor to promote VSMC calcification

**Fig. 7:**
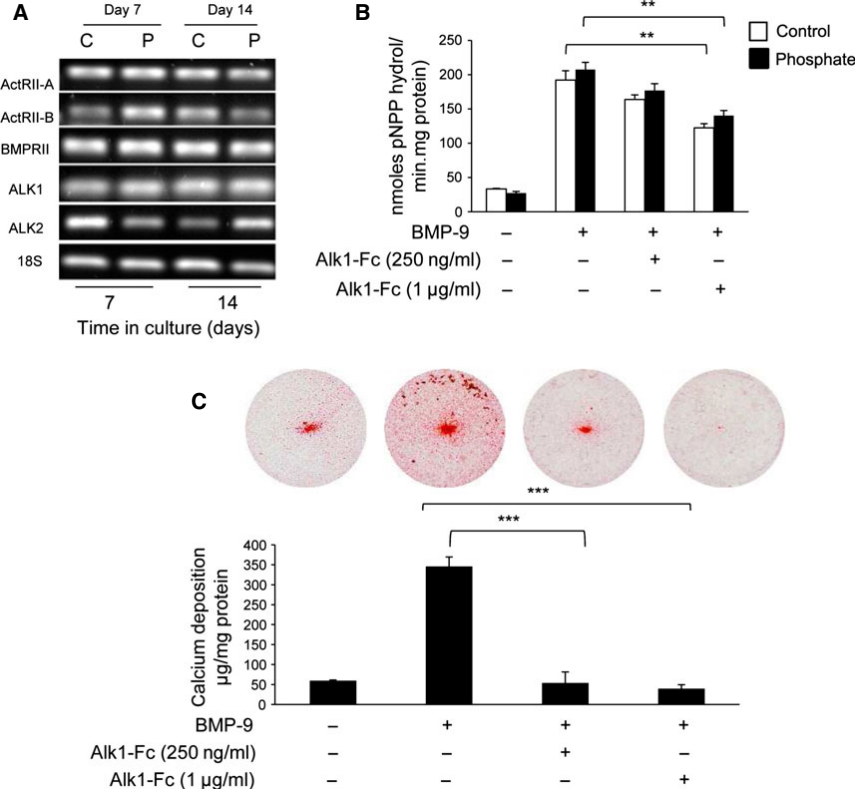
Effect of ALK-1 inhibition on bone morphogenetic protein (BMP-9)-induced VSMC calcification. (**A**) Expression of BMP receptors in VSMCs cultured in high phosphate (3 mM P_i_; P) or control (1 mM P_i_; C) medium for 7 and 14 days. (**B**) Quantification of alkaline phosphatase activity (mean moles pNPP hydrol/min/mg protein in VSMCs incubated with BMP-9 (50 ng/ml) in high phosphate (3 mM P_i_; filled bar) or control (1 mM P_i_; white bar) medium in the presence/absence of ALK1-Fc (250 ng/ml and 1 μg/ml) for 4 days (*n* = 5). (**C**) Calcium content was quantified by HCL leaching (μg/mg protein) in VSMCs incubated with BMP-9 (50 ng/ml) in high phosphate (3 mM P_i_) in the presence/absence of the ALK1-Fc (250 ng/ml and 1 μg/ml; *n* = 4). Results are presented as mean ± SEM. **P < 0.01; ***P < 0.001.

RT-PCR revealed that both type I and type II BMP receptors (*ALK1, ALK2*, *BMPR-II*, *ActR-IIA* and *ActR-IIB)* are expressed in cultured murine VSMCs (Fig. 7A). It has been reported that BMP-9 prefers to bind with ALK1 [22], therefore we next examined whether BMP-9-induced ALP activity and calcification of VSMCs through the ALK1 receptor. ALK1-Fc (1 μg/ml), a soluble chimeric protein which competitively binds ALK1 ligands [23], significantly inhibited BMP-9 (50 ng/ml) induced ALP activity (33%; *P* < 0.01; Fig. 7B) and markedly reduced the pro-calcificatory actions of BMP-9 on VSMCs (85%; *P* < 0.001; Fig. 7C). These data are the first to demonstrate that BMP-9 signals *via* ALK1 to promote the osteogenic differentiation and matrix calcification of VSMCs.

### Intracellular signalling mechanisms mediating BMP-9 induced VSMC calcification

**Fig. 8:**
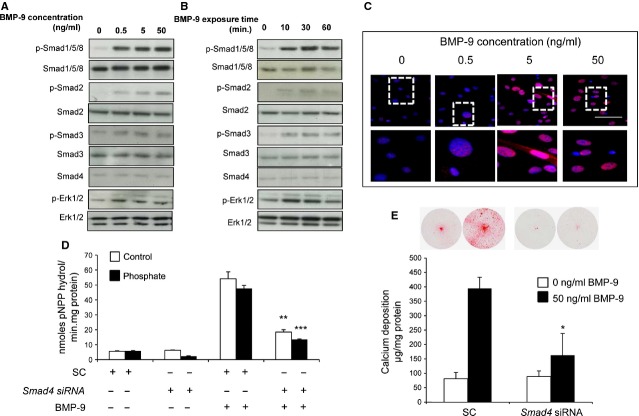
Bone morphogenetic protein (BMP-9) induces VSMC calcification through activation of the Smad signalling pathway. Effect of (**A**) BMP-9 concentration (0.5–50 ng/ml) and (**B**) BMP-9 (50 ng/ml) exposure time (10–60 min) on the phosphorylation (p) of Smad1/5/8, Smad2, Smad3 and Erk1/2 compared with total Smad1/5/8. (**C**) BMP-9-induced (0.5–50 ng/ml) nuclear translocation of phosphorylated Smad1/5/8, (Red). Areas within white markings are shown under increased magnification. (**D**) Quantification of alkaline phosphatase activity (mean moles pNPP hydrol/min./mg protein in VSMCs transfected with *Smad4* siRNA or Scramble control and incubated with BMP-9 (50 ng/ml) in high phosphate (3 mM P_i_; filled bar) or control (1 mM P_i_; white bar) medium for 4 days (*n* = 5). (**E**) Calcium content was visualized with alizarin red staining and quantified by HCL leaching (μg/mg protein) in VSMCs transfected with *Smad4* siRNA or Scramble control and incubated with BMP-9 (50 ng/ml) in high phosphate (3 mM P_i_; *n* = 3). Results are presented as mean ± SEM. *P < 0.05; **P < 0.01; ***P < 0.001 compared with corresponding Scramble control; scale bar = 100 μm.

To investigate the downstream intracellular signalling pathways, VSMC were treated with BMP-9, and activation of Smads and Erk1/2 were assessed by western blotting and immunofluorescent staining. BMP-9 dramatically induced the phosphorylation of Smad1/5/8 and this phosphorylation was observed with concentrations as low as 0.5 ng/ml and reached maximum at 50 ng/ml. The translocation of p-Smad1/5/8 to the nucleus was also observed following BMP-9 treatment (50 ng/ml). Furthermore, the phosphorylation of Smad2, Smad3 and Erk1/2 was weakly induced by BMP-9 (Fig. 8A–C). Smad4 activation was not induced by BMP-9 at any concentration tested.

Smad1/5/8, Smad2 and Smad3 form complexes with the common-partner Smad, Smad4. Transfection of VSMCs with *Smad4* siRNA resulted in an 80% reduction of *Smad4* mRNA (*P* < 0.001) with a comparable decrease in protein expression at 48 hrs post-transfection, which was sustained to 96 hrs (Fig. S1A and B). While short-term exposure of VSMCs to BMP-9 (10–60 min.) did not alter Smad4 expression, transfection of VSMCs with *Smad4* siRNA significantly inhibited BMP-9-induced ALP activity (72%; *P* < 0.001; Fig. 8D) and markedly reduced the pro-calcificatory actions of BMP-9 on VSMCs (61%; *P* < 0.001; Fig. 8E). These results are the first to show that BMP-9 signals through Smad4 to promote the osteogenic differentiation and calcification of VSMCs.

## Discussion

This study expands our current knowledge of the importance of BMP signalling in vascular calcification. Here, we provide the first evidence to suggest that BMP-9, one of the most osteogenic BMPs, also contributes to this pathological process.

Investigations into the natural history of vascular calcification in children with CKD have provided evidence that vessel wall calcification begins pre-dialysis, with factors specific to the dialysis milieu triggering accelerated calcification [3,24]. Indeed, a number of uraemic toxins have been reported to accelerate vascular calcification *in vitro*, including oxidized proteins [25,26], phosphorus [27,28], lipids [29], parathyroid hormone-related peptide [30] and calcitriol [31]. Following reports that BMP-9 circulates under a biologically active form [22] and is present in human serum [32], we sought to compare BMP-9 levels in pre-dialysis and dialysis serum from children. Remarkably, BMP-9 was notably elevated in serum from dialysis patients. This may reflect increased BMP-9 production and/or reduced clearance in the presence of severe kidney failure. While our *in vitro* studies suggest that the circulating active form of BMP-9 is locally activated in VSMCs, additional systemic sources cannot be discounted. Interestingly, the most pronounced effects of BMP-9 administration *in vitro* were seen at concentrations 100-fold higher than the serum BMP-9 concentrations noted in CKD dialysis patients. This suggests potential enhancement of BMP-9 biological activity *in vivo*. BMP-9 may therefore represent a novel kidney marker that predicts progression to a major renal end-point. These findings are further supported by previous reports highlighting disturbed concentrations of additional BMPs, including BMP2 and BMP7, in dialysis patients [33–35].

Our subsequent *in vitro* investigations revealed that VSMCs cultured with recombinant BMP-9 showed increased expression of *Runx2*, *Osterix*, *Akp2* and *PiT-1*, which are recognized regulators of osteoblastic differentiation and matrix mineralization of VSMCs [4,18,36]. Furthermore, BMP-9 directly regulates the matrix mineralization of VSMCs, through an ALP dependent mechanism involving ALK1 receptor binding. ALP has been previously identified as a key promoter of vascular calcification, *via* its ability to hydrolysis the calcification inhibitor pyrophosphate [37]. In addition, our data suggest that this process may be driven by increased PiT-1 expression in the presence of elevated phosphate. These findings support previous data showing that ALK1-Fc inhibits BMP-9-mediated ALP expression in C2C12 cells [38], and disclose for the first time the use of the ALK1-Fc chimera to mediate significant loss of VSMC calcification.

Mechanistically, our data suggest that BMP-9 stimulates the phosphorylation of Smad1/5/8, which form a heterodimeric complex with Smad4 in the nucleus and induces VSMC calcification. However, potential synergy between BMP-9 and other TGFβ superfamily members expressed by VSMCs in response to phosphate treatment cannot be discounted. This study highlights the Smad signalling pathway as a hub in driving BMP-9 induced gene expression changes to initiate aortic calcification. Our results compliment recent clinical studies showing increased Smad1/5/8 phosphorylation in atherosclerotic lesions and calcified aortic valves [39,40] and data further suggest that activation of Smad2, Smad3 and Erk1/2 pathways may refine the effects of BMP-9 on VSMC calcification.

In conclusion, we have undertaken clinical analyses, in conjunction with *in vitro* VSMC calcification studies, to provide fundamental insights into the role of BMP-9 as a potent osteogenic inducer of vascular calcification. BMP-9 therefore appears to play a critical role in vascular calcification. Further translational studies involving human tissues are therefore required to assess whether BMP-9 may represent a novel potential therapeutic target for clinical intervention.

## Supporting information

**Figure S1** Knock-down efficiency of *Smad4* siRNA.

**Table S1** Primer pairs used for PCR analysis.
