# Supplementary figures and images for: BMP-9 regulates the osteoblastic differentiation and calcification of vascular smooth muscle cells through an ALK1 mediated pathway
Source: J Cell Mol Med. 2014 Oct 9;19(1):165–74. doi: 10.1111/jcmm.12373 (PMC4288360; doi:10.1111/jcmm.12373)

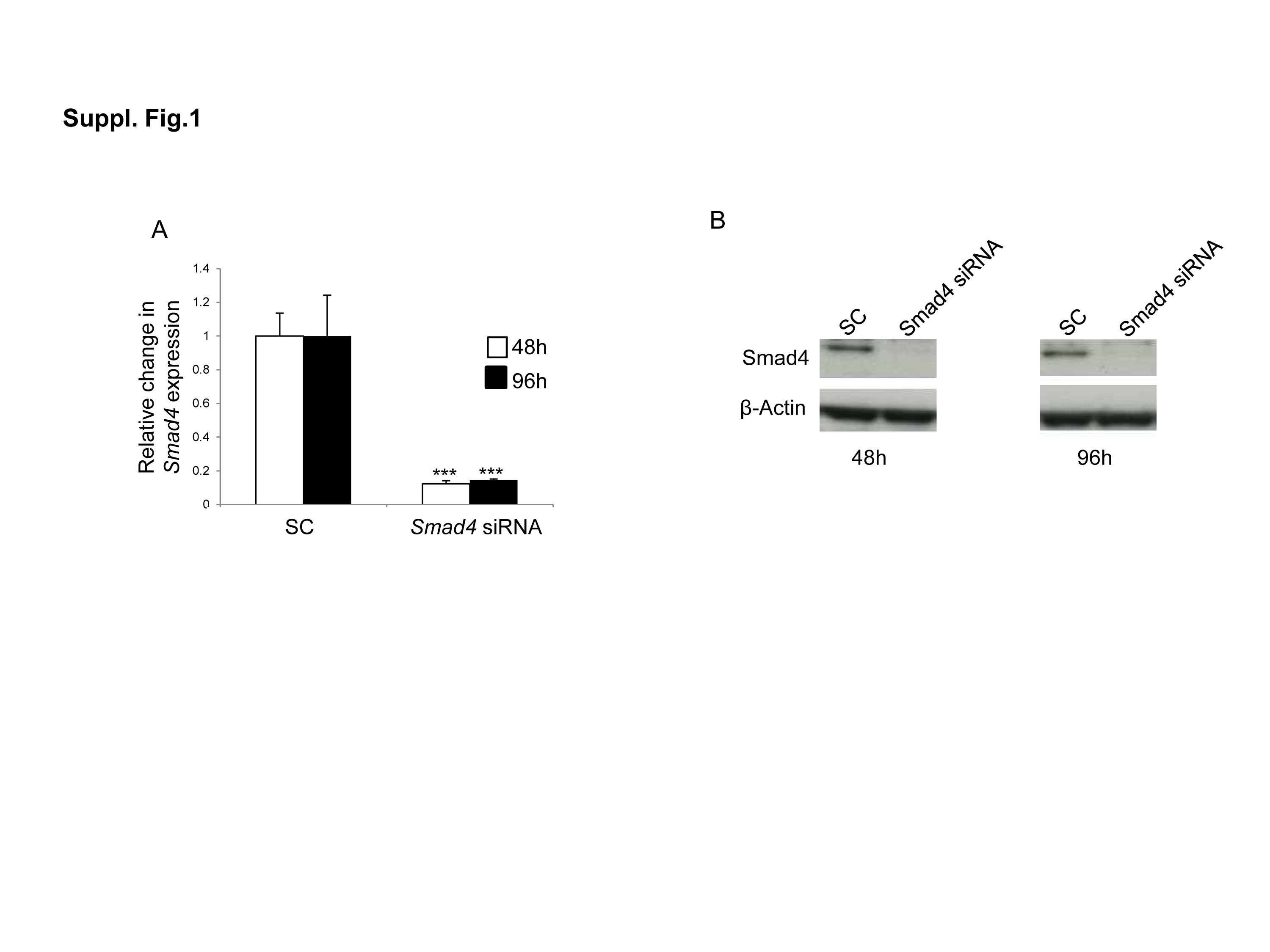

Supplement: Supplementary file 1 — Figure S1 Knock-down efficiency of Smad4 siRNA. [file jcmm0019-0165-sd1.tif]
