# Supplementary material for: BMP-9 regulates the osteoblastic differentiation and calcification of vascular smooth muscle cells through an ALK1 mediated pathway
Source: J Cell Mol Med. 2014 Oct 9;19(1):165–74. doi: 10.1111/jcmm.12373 (PMC4288360; doi:10.1111/jcmm.12373)
Supplement: Supplementary file 2 — Table S1 Primer pairs used for PCR analysis. [file jcmm0019-0165-sd2.docx]

| **Gene** | | | **Primer sequence** |
| --- | --- | --- | --- |
| Pit-1 | Forward  Reverse | 5’ CAC TCA TGT CCA TCT CAG ACT 3’  5’ CGT GCC AAA GAA GGT GAA C 3’ | |
| ALP (Akp2) | Forward  Reverse | 5’ GGG ACG AAT CTC AGG GTA CA 3’  5’ AGT AAC TGG GGT CTC TCT CTT T 3’ | |
| Runx2 | Forward  Reverse | 5’5’ACC ATA ACA GTC TTC ACA AAT CCT 3’  5’ 5’CAG GCG ATC AGA GAA CAA ACT A 3’ | |
| Mgp  ALK1  ALK2  ActRIIA  ActRIIB  BMPRII | Forward  Reverse  Forward  Reverse  Forward  Reverse  Forward  Reverse  Forward  Reverse  Forward  Reverse | 5’ CTG GCA ACC CTG TGC TAC 3’  5’ CAG GCT TGT TGC GTT CC 3’  5’ AAG CCT TCC AAG CTG GTG AA 3’  5’ GCA GAA TGG TCT CTT GCA GTG T 3’  5’ GGA GTA ATG ATC CTT CCT GTG C 3’  5’ TCT TAC ACG TCA TCT TCC CCT G 3’  5’ GTT GAA CCT TGC TAT GGT GAT AA 3’  5’ AAT CAG TCC TGT CAT AGC AGT TG 3’  5’ CAC AAG CCT TCT ATT GCC CAC AG 3’  5’ CAT GTA CCG TCT GGT GCC AAC 3’  5’ TGG CAG TGA GGT CAC TCA AG 3’  5’ TTG CGT TCA TTC TGC ATA GC 3’ | |
| Gapdh  Bmp2;  Bmp9;  Osterix;  Sost; Smad4 | | | **Unknown, purchased commercially from Primer Design.***  **Unknown, purchased commercially from Qiagen.*** |

**Supplementary Table 1: Primer pairs used for PCR analysis.**
